# Farmers knowledge and perception on maize stem borers and their indigenous control methods in south western region of Cameroon

**DOI:** 10.1186/s13002-015-0061-z

**Published:** 2015-11-09

**Authors:** Esther Obi Oben, Nelson Neba Ntonifor, Sevilor Kekeunou, Martin Nkwa Abbeytakor

**Affiliations:** Department of Animal Biology and Physiology, Faculty of Science, University of Yaounde 1, P. O. Box 812, Yaounde, Cameroon; Department of Zoology and Animal Physiology, Faculty of Science, University of Buea, Buea, Cameroon; Association of South West Agriculturist Rural Development Project (ASWA-RUDEP), Buea, South West Region Cameroon

**Keywords:** Pest management systems, Farmers’ perception, Indigenous knowledge, Plant-based insecticides, Maize stem borers

## Abstract

**Background:**

Agriculture is a major contributor to the Gross Domestic Product (GDP) of Cameroon, The South West region of Cameroon is known for its potential in the production of major agricultural commodities, but farmers’ yields from various speculations are low, dwindling over time due to some major constraints. Maize production is hampered by adverse socio-economic factors, several pests and diseases as well as high rainfall with low solar radiation. Lepidopterous maize stem borers are a major threat to increase maize production. Therefore we hypothesized that the farmers of the South West region: (1) also perceived stem borers as an important pest of maize; (2) they have their own indigenous methods of control; (3) they use chemical pesticides because they have no alternative, but would prefer plant materials if these were standardized.

**Methods:**

A semi-structured questionnaire survey was administered in four villages: Maumu, Lower Bokova, Ekona and Bonduma. A total of 151 (male and female) farmers were randomly interviewed to document farmers’ perception on stem borers, and their use of indigenous knowledge to manage key pests of maize.

**Results:**

Stem borers were present throughout the maize growing areas in the Fako division and ranked as one of the most important pests of the crop. Most farmers (82.1 %) perceived that stem borers caused significant damage on maize and were responsible for yield reductions in the crop. The increased impact of these pests was due to improper/untimely use of expensive conventional insecticides given the lack of a cheaper alternative method of control. About 50 % of respondent admitted not having any indigenous knowledge of stem borer control, while only 20 % had tried plant products. The most relevant indigenous stem borer control was the use of wood ash. Most (90 %) of the respondent would prefer plant-based insecticides in future because they are safer, cheaper and readily available.

**Conclusions:**

Farmers’ knowledge would contribute in understanding the activities of stem borers and use of plant insecticides. Research is therefore needed to standardize the methods of using plant-based products and also identify the active ingredients of these plants to ensure their effectiveness against maize stem borers and other pests.

## Background

Agriculture is the primary production sector that contributes to the Gross Domestic Product (GDP) of Cameroon [1]. It is also the main source of employment in the country. Maize production is the main source of income for more than three million small-scale farmers in Cameroon. Though the government subsidizes maize production, this support falls far short of farmers’ needs due to the myriads of production constraints including persistent pest problems such as stem borers.

Lepidopterous stem borers seriously limit potentially attainable maize yields by infesting the crop throughout its growth stages from seedling to maturity [2]. In Cameroon, maize is grown across all agro-ecological zones, from sea level to the highlands at 2000 m a.s.l., and the stem borers are present in all these zones. However, the pest densities and plant damage vary greatly between fields [3]. The most important species that reduce maize yield in West and Central Africa are the pink stalk borer, *Sesamia calamistis* Hampson (Lepidoptera: Noctuidae), the African sugarcane stalk borer, *Eldana saccharina* Walker (Lepidoptera: Pyralidae) and the maize stalk borer, *Busseola fusca* Fuller (Lepidoptera: Noctuidae) [4–6]. Yield losses of food grain in areas with severe borer problems vary between 10-70 % [7–10]. In most of Cameroon, these stem borers are often controlled using conventional insecticides. However, these Synthetic insecticides are unacceptable since they may lead to problems of toxic residues, health and environmental hazards [9, 11] when inappropriately used. As stem borers burrow into the stem, they are often protected from contact insecticides [10]. Various methods of cultural control of stem borers in Africa have been reviewed [12–15], these are most relevant and economical to African resource-poor farmers. Though many of these methods are labor intensive, they have little adverse environmental effects and are readily applicable without extra investment in expensive equipment

In Tanzania farmers have greatly relied on indigenous knowledge and/or plant pesticides to meet their daily needs [16]. This knowledge is most relevant to the rural poor and marginalized population. Several indigenous plant based pest management options used for the control of field and storage insect pests have been identified by research in parts of Tanazania [17]. The studies indicated that botanical formulations reduced stem-borer load by more than 55 % and increased maize yield by more than 60 % compared to the control. Over several decades indigenous knowledge practices have been incorporated into scientific knowledge for development and conservation of natural resources [18]. Hegazy et al. [19] and Mugisha-Kamatenesi et al. [20], found that if thoroughly investigated, the current knowledge gained from indigenous plant species may provide more goods and services for local use. In many parts of Africa, derivatives of indigenous plants like *Piper guineense* and *Tephrosia vogelii* gained attention because of their insect pest control potential [21, 22]. More so, *Jatropha curcas* (native to the American tropics, but widely distributed from Senegal to Cameroon) is also widely used for pest control. Entire or powdered fruits of *Piper* spp*.* have insecticidal and/or repulsive effects against many pests [23–25].

Despite the enormous potential that has existed for generations, plant based indigenous pest control practices have remained largely unexploited due to limited research intervention and resources committed. However, current interest in reducing environmental contamination and global warming are serving as added impetus for the re-evaluation and intensification of environmentally friendly and cost-effective pest management technologies such as the use of traditional botanical pest control agents [26]. Many studies carried out in parts of Africa found that plant derived ash including those of wood and cocoa pod increased P, K, Ca, Mg status of soil and pH and yield of vegetables, rice, millet and maize [27–29]. Farmers’ knowledge of various constraints varies qualitatively/quantitatively depending on their interest in the subject, the environment, and its relevance to their lives. Use of indigenous and plant-based insecticides has been greatly neglected in Fako division and this may partly explain why farmers rely solely on synthetic pesticides. In order to improve food security and alleviate poverty in this region, indigenous pest control measures need to be documented and scientifically validated. Their methods of use also need to be standardized in order to popularize these age-old practices.

Therefore, these studies were conducted to investigate farmers’ knowledge and perception on maize stem borers and their indigenous control methods in South Western region of Cameroon. We hypothesized that the farmers: (1) perceived stem borers as important pest of maize; (2) they have their own indigenous methods of control and only (3) use chemical pesticides because they have no alternative, but would prefer plant material if these are standardized since they are safer and cheaper.

We therefore sought to know whether or not farmers in the South West Region of Cameroon know about maize stem borers, if they rate them as important pests of maize and how they combat this problem. Farmers were also asked whether or not they used any indigenous methods and/or plant-based products against stem borers, if they could recognize the plants used and how effective these methods were vis-à-vis chemical/synthetic products as well as whether they will prefer to use indigenous or conventional methods to mitigate their maize pest problems in future?

We also sought to know if there were differences in knowledge and practices in controlling maize pest problems between men and women as well as among the different villages studied.

## Materials and methods

### Study site

The survey was conducted in the rainforest agroecological zone of Cameroon. A total of 151 farmers from four villages (Maumu and Ekona for Muyuka subdivision and Lower Bokova, and Bonduma for Buea sub division) in the Fako division of the South West Region were interviewed. Farmers were selected on the bases that each has been involved in maize cultivation for at least one year and were willing to participate in the study. The villages used in the study are in Buea (4°08’ 036”N, 9°25’ 826”E; 573 m asl) with rich volcanic rocky soils and temperature ranges of 20-25 °C and Muyuka (4°150’ 45”N, 9°28’ 431”E; 599 m asl) with sandy soil and high temperatures ranges from 20–28.1 °C, an altitude of 378 m sub-divisions. The location of these villages in a predominantly agrarian area and gender heterogeneity of the participants was a strong driving force on the farmers’ perceptions. Buea is more cosmopolitan, with mountainous rich volcanic soils and favorable climatic conditions for maize cultivation. Muyuka, being warmer, favors the rapid buildup of pest populations. The farming community of the Maumu village in Muyuka is inaccessible due to poor road infrastructures and heavy rains, particularly during the rainy season (June to September). The poor roads prevent proper functioning of markets and lack of agricultural inputs. Most of the farmers being females have no direct contact with extension workers. The main food crops in the region are maize *Zea mays*, cassava *Manihot* spp, cocoyam *Colocasia esculentum*, groundnuts *Arachis hypogaea*, beans *Phaseolus vulgaris*, banana/plantains *Musa* spp, with vegetables and few spices as secondary crops while oil palm *Elaeis guineensis*, cocoa *Theobroma cacao* and coffee are the main cash crops.

### General characteristics of respondents

Generally, small-scale (subsistence) farming (86.09 %) was the primary economic activity of most of the respondents while the remaining, (9.93 %) practiced farming as a part time job. Within the different villages, 100 % of respondents in Maumu; 40.63 % in Bonduma; 97.73 % in Ekona and 97.50 % in Lower Bokova respectively practiced farming as their main occupation. Maize farming experience ranged from 1–50 years. All of the respondent (100 %) grow food crops, 32.45 % of these also grow cash crops, while 13.33 % kept animals in addition. In all, 76.82 % of the respondents grow their maize in mixed cropping system, 17.22 % in mono cropping while 5.96 % practiced both.

Besides the growing of food crops, most of the farmers in Muyuka subdivision also grew cash crops, especially coffee. Most (94.04 %) of the farmers plant maize twice a year, while 2.65 % and 3.31 % respectively grow maize once and three times a year. Most (92.05 %) of the harvested maize is used for household feeding; while 7.95 % is for sale or animal feed. The majority of the respondents (58.94 %) had completed primary education, 16.56 % had no formal education while 24.51 % had secondary and pre-university education. Within the four villages, 28.13 % in Bonduma, 61.36 % in Ekona, 62.50 % in Lower Bokova and 80.00 % in Maumu of the respondents had completed primary education. The study revealed that more farmers in Buea were educated, and involved in business than those in Muyuka. In Muyuka most of the farmers had no formal education; therefore farming was their main source of living (Table 1).

**Table 1:** Variables showing the significant differences between Muyuka and Buea subdivisions

	Harvest quantity (KG)	Education (None)	Occupation (Business)	Occupation (Farming)	Crop grown (coffee)	Cropping system (Both)	Main pest problem (Snails)	Type of pesticide use (Cypercal 12)	Period of treatment (september)	Future preference (conventional)
Buea	1206 ± 1840.80	28 (7)	88.89 (8)	40 (52)	30.6 (15)	44.4 (4)	10 (1)	91.7 (22)	76.9 (20)	25 (10)
Muyuka	478.99 ± 1040.49	72 (18)	11.11 (1)	60 (78)	69.4 (34)	55.6 (5)	90 (9)	8.3 (2)	23.1 (6)	75 (30)
X^2^value or Z-value*	3.61*	4.8	5.44	5.2	7.4	0.11	6.4	16.7	7.5	10
*P*-value	0.0003	0.03	0.02	0.022	0.007	0.008	0.01	0.0001	0.006	0.002

Z is a Wilcoxon two sample test value

### Survey

A semi-structured questionnaire was used in the survey. A total of 151 farmers (72.85 % females and 27.15 % males) 35 in Maumu, 40 in Lower Bokova, 44 in Ekona and 32 in Bonduma, were interviewed separately within their farming areas or around their residence. Farmers were selected on the bases that each has been involved in maize cultivation for at least one year and were willing to participate in the study. Interviews were done in English or local language (pidgin) with the assistance of local agricultural extension workers.

The questionnaire sought to know: (a) the kind of indigenous methods and plant products used by farmers for maize stem borer control and their main constraints, as well as their knowledge on stem borer problems (b) if they use chemical/synthetic products to control pests, their names, the frequency of use and the constraints linked to their use; (c) whether they had contacts with agricultural extension workers, and their future preference between indigenous and conventional methods in dealing with pests and disease problems in maize fields. Data were also collected on the socio-economic characteristics of respondents.

### Statistical analysis

For each variable, statistical comparison between the two sexes, the two locations and four villages were done based on the procedure of the software SAS (‘Statistical Analysis Systems’ version 9.1). The frequencies of respondent were also analysed with Chi-square test using PROC PREQ while the Kruskal-Wallis and Wilcoxon two sample tests using the ‘Nonparametric One Way’ (‘NPAR1WAY WILCOXON’) procedure were used in the absence of normality to compare the means of quantitative variables. All probabilities were appreciated at the 5 % confidence level.

## Results

### Main constraints and major pest problems in maize production

Participants revealed that the major constraints to increased maize production were: land, labour, finance as well as pest and diseases (Table 2). Pests and diseases were the major constraints (85.43 %), followed by finances (55.63 %), land (37.09 %) and labour (28.48 %). There were significant differences between males and females in each village with regard to pests and disease problems. In Maumu, 100 % of the males and 48.15 % of females considered pest and disease as a problem. The same trend was observed in Ekona (100 % and 93.75 %) and Lower Bokova (88.10 % and 70.83 %), where males and females respectively considered pest and diseases as their major constraint. On the contrary, in Bonduma more females (94.12 %) than males (53.33 %) considered pests and diseases as main constraints.

**Table 2:** Percentage (±i) respondent relative to barriers to increase maize production in Fako Division of South Western Cameroon

All						Overall
Major constraints	Maumu (*n* = 35)	Lower Bokova (*n* = 40)	Ekona(*n* = 44)	Bonduma (*n* = 32)	*P*-values	
Land	42.9 ± 7.9a	22.5 ± 6.7b	38.6 ± 7.8a	46.9 ± 8.0a	0.0015	37.1 ± 7.7
Labour	22.9 ± 6.7b	32.5 ± 7.5a	18.2 ± 6.1b	43.8 ± 7.9a	<0.0001	28.5 ± 7.2
Finances	48.6 ± 8.0a	47.5 ± 7.9a	63.6 ± 7.7a	62.5 ± 7.7a	0.1665	55.6 ± 7.9
Pests and diseases	97.1 ± 2.7a	80.0 ± 6.4b	88.6 ± 5.1b	75.0 ± 6.9b	<0.0001	85.4 ± 5.6
Females						
Land	55.6 ± 7.9a	8.3 ± 4.4c	35.71 ± 7.64b	64.71 ± 7.62a	0.0221	39.1 ± 7.9
Labour	18.5 ± 6.2c	29.2 ± 7.3b	14.29 ± 5.58c	47.06 ± 7.96a	<0.0001	23.6 ± 6.8
Finances	48.2 ± 7.9a	41.7 ± 7.9a	61.90 ± 7.75a	52.94 ± 7.96a	0.5673	52.7 ± 7.9
Pests and diseases	48.2 ± 7.9b	70.8 ± 7.3a	88.10 ± 5.16a	94.12 ± 3.75a	<0.0001	87.3 ± 5.3
Males						
Land	0	43.8 ± 7.9b	100a	26.7 ± 7.1c	0.0191	31.7 ± 7.4
Labour	37.50 ± 7.72b	37.5 ± 7.7b	100b	40 ± 7.8b	0.2743	41.5 ± 7.9
Finances	50 ± 7.98c	56.3 ± 7.9c	100a	73.3 ± 7.1b	0.0858	63.4 ± 7.7
Pests and diseases	100a	93.8 ± 3.9a	100a	53.3 ± 7.9b	<0.0001	80.5 ± 6.3

Zones not sharing a common letter in a row are significantly different at *P* = 0.05. ‘p-value’ is the significance level of Kruskal-Wallis (*NA* no significance, *P* >0.05)

Stem borers (82.12 %) ranked the highest among the major field pest and disease problems of maize in the study areas. Male and female perceptions were significantly different in all four villages (Table 3). In Maumu, 100 % of female farmers considered stem borers as their major field pest, as against 75 % males. In Bonduma and Ekona, the trend was the same, while in Lower Bokova more males (100 %) than females (95.83 %) perceived stem borer as a major threat to maize production. Some of the farmers described that the larvae of the pest bore into the stems and cobs, causing wilting of the plants. Cobs with borer tunnel holes when taken to the market sold at cheaper prices, due to their lower aesthetic values.

**Table 3:** Percentage (±i) respondent relative to major pests as barriers to increase maize production in Fako Division of South Western Cameroon

All						Overall
Major pests	Maumu (*n* = 35)	Lower Bokova (*n* = 40)	Ekona (*n* = 44)	Bonduma (*n* = 32)	*P*-values	
Stem borers	94.3 ± 3.7a	97.5 ± 2.5a	70.5 ± 7.3b	65.6 ± 7.6b	<0.0001	82.1 ± 6.1
Weevils	5.7 ± 3.7b	10.0 ± 4.8b	13.6 ± 5.5b	40.6 ± 7.8a	<0.0001	16.6 ± 5.9
Snails	2.9 ± 2.7b	0	18.2 ± 6.2a	3.1 ± 2.8b	<0.0001	6.6 ± 3.9
Thieves (Human)	0	7.5 ± 4.2	0	0	<0.0001	1.9 ± 2.2
White grubs	2.9 ± 2.7b	0	13.6 ± 5.5b	37.5 ± 7.7a	<0.0001	11.9 ± 5.2
Birds	2.9 ± 2.7b	5.0 ± 3.5b	4.6 ± 3.3b	25 ± 6.9a	<0.0001	8.6 ± 4.5
Females						
Stem borers	100a	95.8 ± 3.2a	71.4 ± 7.2b	70.6 ± 7.3b	<0.0001	83.6 ± 5.9
Weevils	0	12.5 ± 5.3b	11.9 ± 5.2b	41.2 ± 7.9a	<0.0001	13.6 ± 5.5
Snails	0	0	16.7 ± 5.9	0	<0.0001	6.4 ± 3.9
Thieves (Human)	0	4.2 ± 3.2	0	0	<0.0001	0.9 ± 1.5
White grubs	0	0	14.3 ± 5.6b	47.1 ± 7.9a	<0.0001	12.7 ± 5.3
Birds	0	8.3 ± 4.4b	4.8 ± 3.4b	17.7 ± 6.1a	<0.0001	6.4 ± 3.9
Males						
Stem borers	75 ± 6.9b	100a	50 ± 7.9c	60 ± 7.8b	0.0003	78.1 ± 6.6
Weevils	25 ± 6.9b	6.3 ± 3.9b	50 ± 7.9a	40 ± 7.8a	0.0010	24.4 ± 6.8
Snails	12.5 ± 5.3b	0	50 ± 7.9a	6.7 ± 3.9b	<0.0001	7.3 ± 4.2
Thieves (Human)	0	12.5 ± 5.3	0	0	<0.0001	4.9 ± 3.4
White grubs	12.5 ± 5.3a	0	100	26.7 ± 7.1a	<0.0001	9.8 ± 4.7
Birds	12.5 ± 5.3b	0	0	33.3 ± 7.5a	<0.0001	14.6 ± 5.6

Zones not sharing a common letter in a row are significantly different at *P* = 0.05. ‘*p*-value’ is the significance level of Kruskal-Wallis (*NA* no significance, *P* >0.05)

Apart from the stem borers, some farmers in Muyuka also considered snails (6.62 %) as a very important pest, especially during the heavy rains (Table 1). Other pest problems included weevils (16.56 %), white grubs (11.92 %), birds (8.61 %) as well as theft (1.99 %). Due to their meager earnings, most farmers also complained of high prices of pesticides as one of their major constraint.

### Indigenous knowledge and efficacy of plant-based control

The survey showed some knowledge on use of indigenous methods for maize pests and diseases control (Table 4). 54.30 % of the total respondents admitted not having any indigenous knowledge, while 45.70 % used indigenous methods to combat stem borers. In Ekona, Bonduma and Maumu, 72.73 %, 43.75 % and 40 % respectively of respondent used indigenous method as against 22.50 % in Lower Bokova. Within each village, males and females had different perceptions on indigenous control methods; 76.91 % of females in Ekona used indigenous control against zero males. In Lower Bokova and Bonduma, more males than females used indigenous control, while in Maumu more females (44.44 %) than males (25 %) used indigenous control.

**Table 4:** Percentage (±i) respondent relative to use of plant-based/indigenous stem borer control in Fako Division of the South Western Cameroon

All						Overall
Control of stem borers		Maumu (*n* = 35)	Lower Bokova (*n* = 40)	Ekona (*n* = 44)	Bonduma (*n* = 32)	
Plant-based products	Yes	31.4 ± 7.4a	0	36.4 ± 7.7a	3.1 ± 2.8b	18.5 ± 6.2
	No	68.6 ± 7.4b	100	63.6 ± 7.7b	96.9 ± 2.8a	81.5 ± 6.2
	*P*-values	0.0280	0	0.0704	<0.0001	<0.0001
Other indigenous methods	Yes	40 ± 7.8b	22.5 ± 6.7b	72.7 ± 7.1a	43.8 ± 7.9b	45.3 ± 7.9
	No	60 ± 7.8a	77.5 ± 6.7a	27.3 ± 7.1b	56.3 ± 7.9a	54.7 ± 7.9
	*P*-values	0.2367	0.0005	0.0026	0.4795	0.2901
Females						
Plant-based products	Yes	37.0 ± 7.7a	0	38.1 ± 7.8a	0	23.6 ± 6.8
	No	62.9 ± 7.70a	100	61.9 ± 7.8a	100	73.4 ± 7.1
	*P*-values	0.1779	NA	NA	NA	<0.0001
Other indigenous methods	Yes	44.4 ± 7.9b	20.8 ± 6.5b	76.2 ± 6.8a	41.2 ± 7.9b	50.9 ± 7.9
	No	55.6 ± 7.9a	79.2 ± 6.5a	23.8 ± 6.8b	58.8 ± 7.9a	49.1 ± 7.9
	*P*-values	0.5637	0.0043	0.0007	0.4669	0.8488
Males						
Plant-based products	Yes	12.5 ± 5.3	0	0	6.7 ± 3.9	4.9 ± 3.4
	No	87.5 ± 5.3a	100	100	93.3 ± 3.9a	95.1 ± 3.4
	*P*-values	0.0339	NA	NA	0.0008	<0.0001
Other indigenous methods	Yes	25 ± 6.9b	25 ± 6.9b	0	46.7 ± 7.9a	31.7 ± 7.4
	No	75 ± 6.9a	75 ± 6.9a	100	53.3 ± 7.9b	68.3 ± 7.4
	*P*-values	0.1573	0.0455	NA	0.7963	0.0191

Zones not sharing a common letter in a row are significantly different at *P* = 0.05. ‘*p*-value’ is the significance level of Kruskal-Wallis (*NA* no significance, *P* >0.05)

Only 18.54 % of farmers in this region had any knowledge on the use of plant derivatives in place of chemicals, while 81.46 % have never used any plant product as control against stem borers. However, 37.04 % females used plants as against 12.50 % of males in Maumu, while 38.10 % used plants in Ekona. In Lower Bokova, males and females did not use plants, while in Bonduma 6.67 % of males used plant products. The use of plant-based products was low in some villages because most of the farmers depended on synthetic (Table 4). In Ekona, Lower Bokova, Maumu, Bonduma, 63.64 %, 100 %, 68.57 % and 96.88 %, respectively of respondents do not used any plant materials for control.

Some respondent used wood ash as an indigenous control method (Ekona), and rated it to be very effective. The wood ash was sometimes mixed with Mocap (ethopropos), which is a synthetic product. The only problem was that most of them could not recognize the particular plant or the parts of the plant being used or even the proportion and formulations.

### Indigenous methods of stem borer control in the Region

**Fig. 1:**
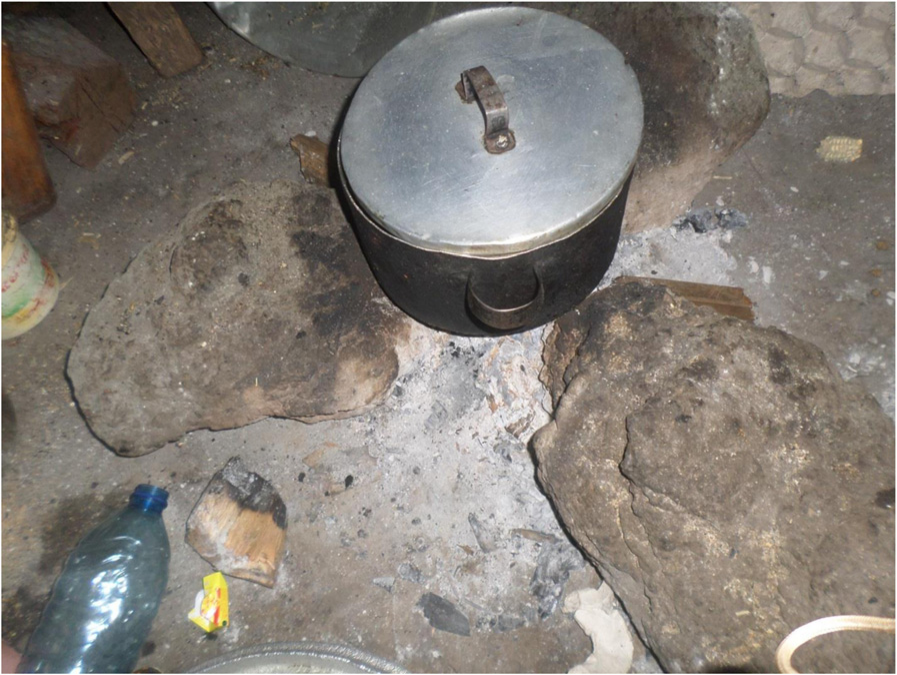
Typical local kitchen with three-stone fire place where wood ash is collected

**Fig. 2:**
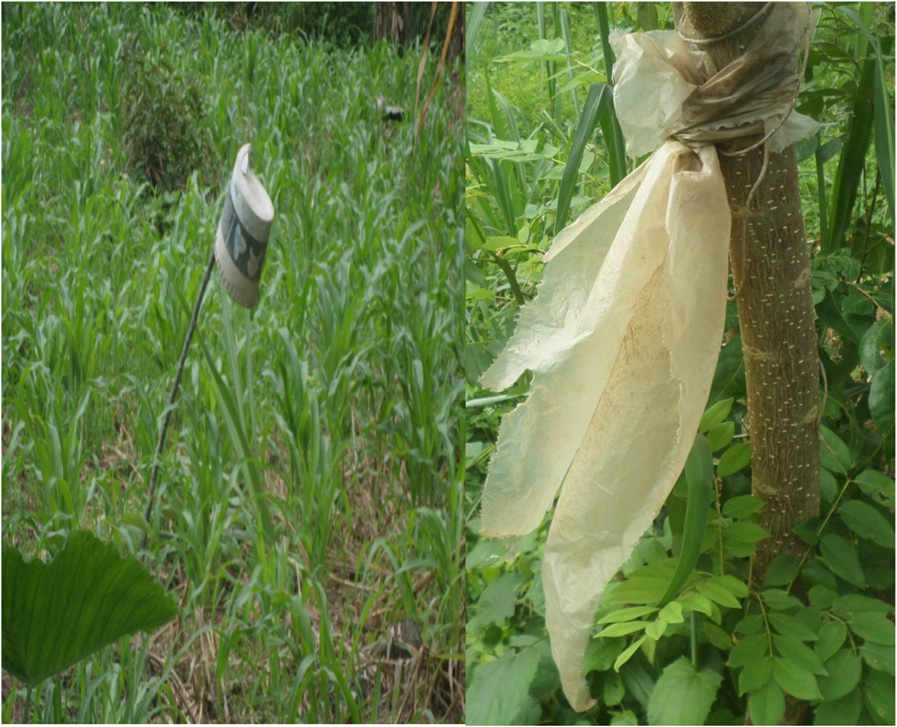
Typical maize farm with torn plastic bags and old plastic buckets tied to keep away birds

**Fig. 3:**
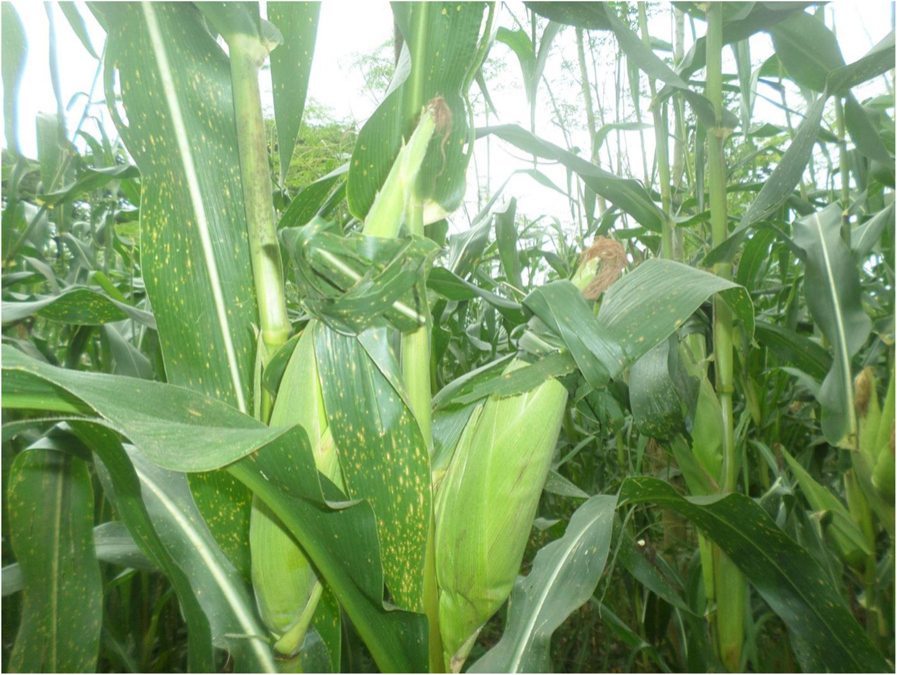
Typical indigenous traditional method for keeping away birds from feeding on the matured maize cobs before harvest

Various indigenous control methods were enumerated by the farmers. The main ingredient in most of them was ash collected from household kitchens or burnt wood (Fig. 1). Some used only the dry wood ash while others mixed fine dry soil with wood ash. Some others mixed their ash with conventional insecticides such as Mocap (ethopropos), Sevin, Gamalin or Kerosene. Some mixed the ash with water or Kerosene and used as sprays. In all of these methods, treatment was applied within the leaf whorl of the plant without any personal protective equipment (PPE). This raises safety concern for persons doing the applications. To keep away other larger insects and birds, farmers hang torn plastic bags, old plastic buckets in and around their fields especially just after planting (Fig. 2). Other farmers just simply let go and face the consequences of their maize being destroyed by these pests thus reducing average yields. Sometimes the birds fed on the cobs when the maize was matured. To remedy such situations, some farmers tied the leaves closest to the cobs around the cob to prevent the birds from feeding on the grains (Fig. 3).

### Use of pesticides

In all four villages surveyed, 70.20 % of respondents used pesticides to treat their maize farms and 29.80 % did not (Table 5). More farmers in Buea used conventional pesticides, especially Cypercal 12 than those in Muyuka; most of their treatments were applied in the month of September

**Table 5:** Percentage (±i) respondent relative to use of pesticides in Fako Division of South Western Cameroon

All					Overall
Conventional methods	Maumu (*n* = 35)	Lower Bokova (*n* = 40)	Ekona (*n* = 44)	Bonduma (*n* = 32)	
Use pesticides	57.1 ± 7.9b	95 ± 3.5a	79.6 ± 6.4a	40.6 ± 7.8b	70.2 ± 7.3
Do not use pesticides	42.9 ± 7.9a	5 ± 3.5b	20.5 ± 6.4b	59.4 ± 7.8a	29.8 ± 7.3
P-values	0.3980	<0.0001	<0.0001	0.2888	<0.0001
Females					
Use pesticides	48.2 ± 7.9b	91.7 ± 4.4a	78.6 ± 6.6a	35.3 ± 7.6b	67.3 ± 7.5
Do not use pesticides	51.9 ± 7.9a	8.3 ± 4.4b	21.4 ± 6.6b	64.7 ± 7.6a	32.7 ± 7.5
P-values	0.8474	<0.0001	0.0002	0.2253	0.0003
Males					
Use pesticides	87.5 ± 5.3a	100	100	46.7 ± 7.9b	78.1 ± 6.6
Do not use pesticides	12.5 ± 5.3b	0	0	53.3 ± 7.9a	21.9 ± 6.6
*P*-values	0.0339	NA	NA	0.7963	0.0003

Zones not sharing a common letter in a row are significantly different at *P* = 0.05. ‘*p*-value’ is the significance level of Kruskal-Wallis (*NA* no significance, *P* >0.05)

More farmers in Ekona (79.55 %) and Lower Bokova (95 %) depended on pesticide compared to Bonduma (40.63 %) and Maumu (57.14 %). In Maumu, more males (87.50 %) than females (48.15 %) used pesticides while in Ekona and Lower Bokova, all males (100 %) used pesticides as against 91.67 % and 78.57 % of females respectively (Table 5).

**Fig. 4:**
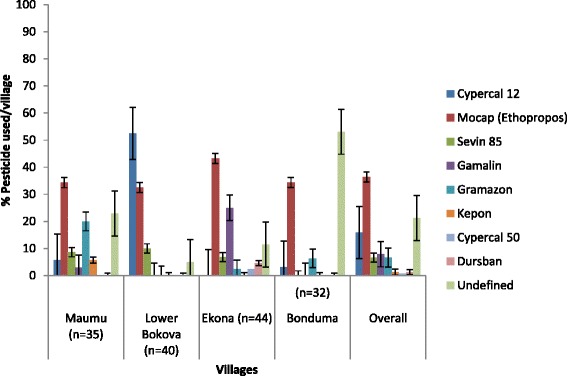
Percentage (±i) respondent relative to main pesticides used in Fako division of the South Western Cameroon

Cypercal 12 was the main pesticide used in Lower Bokova (52.50 %), while Mocap (Ethopropos) was widely used by all farmers, especially in Ekona (43.18 %) (Fig. 4). Mocap (Ethopropos), an organophosphate insecticide was widely used because it was cheaper and easy to get, and is also a contact/repellent product. About 53.13 % of farmers in Bonduma did not use any of the pesticides, though sometimes they got advice from friends on the type of pesticide to use. Some farmers admitted not using any pesticides against stem borers, but sometimes used weed killers, since maize does not tolerate high weed population. Some farmers mixed Mocap (Ethopropos) and wood ash, where the wood ash serves as a carrier substance for the insecticide.

**Fig. 5:**
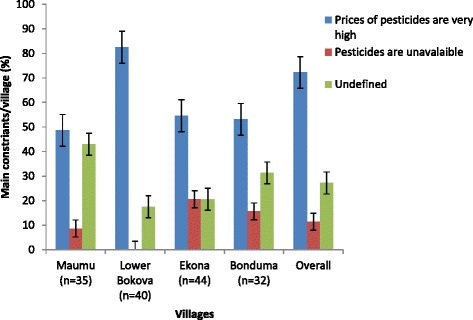
Percentage (±i) respondent relative to main constraints faced in Fako division of South Western Cameroon

**Fig. 6:**
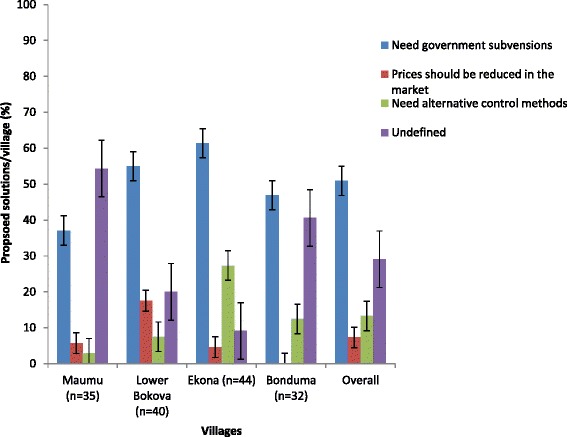
Percentage (±i) respondent relative to possible solutions proposed to constraints in Fako division of South Western Cameroon

The main constraints reported by the farmers regarding the use of pesticides were high prices (72.19 %), while 27.15 % were indifferent (Fig. 5). In Lower Bokova, 82.50 % of farmers complained of high prices, since most of them depended on pesticides. Some of their proposed solutions to these constraints were; need for government subsidies, prices should be moderated in the market and the need for alternative methods of stem borer control (Fig. 6). In Ekona, 61.36 % of farmers believed government subsidies would help resolve the problem of high prices. Others believed pesticides were easier and quicker to administer on farm. More than 50 % of respondents in the four villages proposed government subsidies and price reduction as solutions to the pesticide constraints.

### Farmers’ future preference

Regarding farmers’ future preferences, more of them in Muyuka would prefer the continued use of conventional methods of stem borer control than in Buea. Due to the high level of education and the cosmopolitan nature of Buea, most of the farmers had lost their indigenous knowledge than in Muyuka. They had no basic knowledge on indigenous methods of plants used for pest control.

**Fig. 7:**
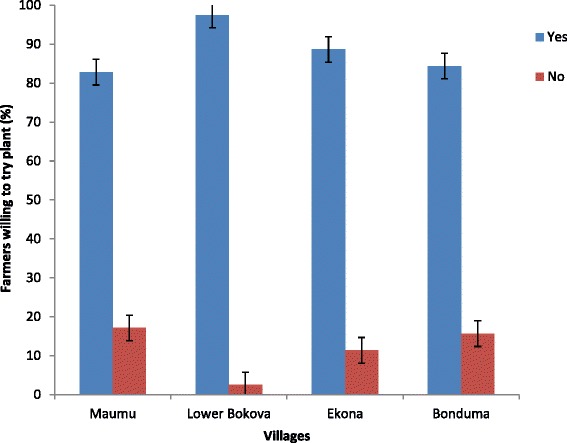
Percentage of maize farmers in Fako division willing to try plant-based insecticides control methods

Most farmers did not have contacts with agricultural extension workers because the few extension workers could hardly visit all the farmers in their areas. This was a major constraint to the farmers since they were not well informed about insecticides and common indigenous methods of pest control. Most of the farmers (88.74 %) expressed interest in the use of plant-based products (Fig. 7). More farmers in Lower Bokova (97.50 %) were willing to try plant-based insecticides than in Bonduma (84.38 %), Ekona (88.64 %) and Maumu (82.86 %). Some of the reasons mentioned were that the plants were safer than synthetic insecticides and also readily available. Others sought to know whether such plants could have long-term effects and were not time-consuming. The remaining 11.26 % did not show any interest in the use of plant products.

**Fig. 8:**
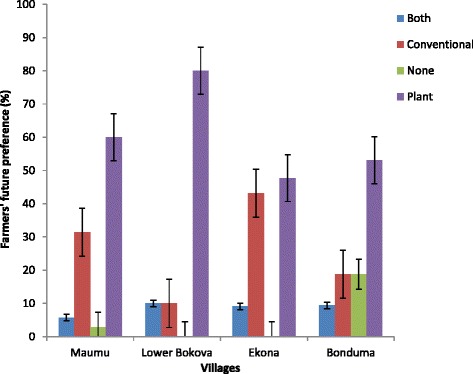
Percentage of maize farmers in Fako division who prefers plant-based insecticide control against conventional products in future

When questioned on their future preferences, 60.26 % declared that they would prefer plant products in place of synthetic insecticides, 8.61 % preferred both synthetic and indigenous, 26.49 % still preferred conventional insecticides while 4.64 % were undecided (Fig. 8). Lower Bokova again showed that more farmers (80 %) would prefer plant-based insecticides as compared to Ekona (47.73 %), Maumu (60 %) and Bonduma (53.13 %). Those who preferred synthetic insecticides believed that if the government could subsidize or reduce the cost of these products in the market, they will be able to afford them. The role of the Ministry of Agriculture is still preponderant as it grants the import authorization and approves the distribution and sales of the agricultural inputs. Some of the study villages are far from the sales point. Consequently, transportation costs rendered costs of inputs out of the reach of many farmers.

## Discussion

Apart from food crops, farmers in Fako division of South West Region of Cameroon also grow cash crops and also rare animals for home consumption. Most of the crops are attacked by field pests during the rainy season when crops are in the vegetative and reproductive stages; this greatly reduces the farmers’ harvests. Most of the pests problems encountered are on cereals, particularly maize. Crop products are stored as food reserves in these areas, while some of the produce are used for income generation. Considering that the maize yields are always low, partly due to pests/diseases and other allied constraints, it has serious implications on their food security needs.

Land, labour, finance, pests and diseases were the most important constraints to maize production in the Fako division of the South West region of Cameroon. As is the case in most parts of Africa [30], most of the smallholders’ farmers in Cameroon are women with two-thirds of their farms being below 2 hectares. Many practice low-resource agriculture based primarily on the use of local resources with modest external inputs. Increased urbanization causes a shift of farmland into urban areas, thus reducing available space for farming [31]. The quantity of available land and farm inputs determine maize output. Similar results were obtained by [32] in the West region of Cameroon, stating that, maize yields were low due to reduced farm sizes, low quality of maize seeds planted, inadequate labour, fertilizer and agrochemicals inputs. Most of the food production burden falls on women and children because of rural urban migration and reduction in active work force wreaked by various diseases and ill-health [33]. Children are sometimes denied the chance to go to school to assist in weeding because of labour scarcity, resulting in low educational performance [34]. Pest and diseases were the major constraints limiting attainable maize yields. In the farmers’ perceptions, pest and diseases were amongst the most important constraints in crops in this region. Most of them showed some degree of awareness of the different insect pests that attack their crops. Similar results were reported by [32] in the forest and humid forest of Cameroon, which corroborates with the findings of [35]. Increase in pest and disease may partly be as a result of increased use of pesticides by larger corporations which makes the pest become resistant and later shift into the untreated fields.

The study showed that the farmers regarded stem borers as important pests of maize in Fako division of the South West Cameroon. Stem borers interfere with the movement of water and metabolites through the plant’s vascular system, which stunts its growth and development. Attacks during the first eight weeks after sowing result in “dead heart” and late damage (beyond eight weeks after sowing) leads to stem lodging. Both types of damage to the crop cause drastic loss in maize yield [36]. The farmers reported an increased damage during the dry season when pest populations are higher. Farmers’ perceived that, the insect pests had economic implications, given that the insects caused significant damage that warranted the implementation of control measures. These perceptions contribute to the understanding of various aspects of the bio-ecology of insect pests [36, 37]. Similar results were also obtained in the humid forest and Western highlands of Cameroon [38–40].

The results indicated that only 45.70 % of the farmers used one indigenous method or another, while 54.30 % depended solely on conventional control methods, which are expensive. Some reported that indigenous methods were time-consuming and they were not sure of the results. Those who used indigenous methods believed they were cheaper and they faced no problems with their use. Cultural/indigenous practices are not expensive for the farmers and do not necessitate in general, supplementary material investments to control insect pests [41]. A large proportion of the farmers believed indigenous control methods are not effective similar to the findings of [41], which stated that the development of traditional/indigenous control methods is very limited.

The results showed that very few farmers were using plants as insect pest control methods in their fields. Farmers perceived plant derivatives could not give the desired results achieved when conventional methods are used. Possibly integrating the use of resistant plants with plant derivatives could be a better option for replacing synthetic chemicals, given that they are simple, economical and important strategies in insect pest control. They are also not dangerous to the environment and are generally compatible with other pest control methods [11, 41]. Farmers’ knowledge and perception of their use can accelerate and facilitate their adoption in the local communities.

More farmers depended on pesticides than on botanical control, although not adequately informed about their proper use similar to studies carried out by the Ministry of Agriculture which showed that more than 42 % of farmers use pesticides [42]. Increased use of pesticides is due to the proliferation and accessibility of unlicensed dealers’ shops that are only out to make money but care less about the consequences of pesticides. Farmers relied mostly on estimations for the amount and concentration required for a given botanical pesticide formulation because most of them are illiterate. Therefore, there might be risk of overdose, since most of them do not have frequent contacts with extension workers. The most mentioned pesticides used by farmers are Mocap (Ethopropos) and Cypercal. The former, being the cheapest and readily available while the latter, classified as “1b” by the World Health Organization (WHO) and qualified as highly dangerous [42] is no longer recommended [43]. Mocap (Ethopropos) is an organophosphate (classified as IA), extremely hazardous to eyes, and the body when inhaled especially as farmers donot use personal protective equipments (PPE) during pesticide applications. Most of these insecticides are also highly dangerous to the environment and pollute water bodies. Substances classified in these category by WHO should not be applied by untrained or inadequately protected people [44–46].

Indigenous control methods were important because most of the farming in these areas are subsistence. Despite the fact that most of the farmers acknowledge the effectiveness of plant-based insecticides, most of them would still prefer conventional products in future if they are affordable. This may be because those who use the indigenous controls and botanicals do not know the right formulations and amounts to apply, as well as the time of application. The main component of this indigenous control was ash from burnt wood collected from local kitchens. Owolabi et al. [29] studied the effect of liming materials such as plant derived ash on maize yield, and found that it increased soil pH and maize yield.

The findings showed that there was heterogeneity in knowledge between the two locations as well as in gender respectively. In most of the responses, the women seemed to perceive the pest incidences with equal importance. Most of the males interviewed were from Lower Bokova (17), followed by Bonduma (14) which are villages in Buea with a higher level of education. While most of the females were found in Ekona (42) and Maumu (27) and farming was their main occupation without any formal education. This confirms why their perceptions were different, as more women than men reported increased incidence of stem borers in their fields. Perception differences in gender have also been observed in Nepal, where men generally used more vague attributes like harmful or harmless, while women were more specific, regarding the depredatory insects [37]. Gender differences in perception may also be due to division of labour, as most of the women in this region spend equal time in fields as well as household tasks while the men are involved in farming and community works.

In absence of a standardized protocol on preparation and application, the indigenous plant-based formulations will have varied efficacies at different times even with the same farmer. Without the standardization of the specific amount of a product used, exposure time and way of preparation and no proper application rate and method, efficacy rating of any pesticide will be compromised. There is therefore need to increase productivity through the development of alternative low-cost plant-derived technologies for fighting pests and diseases in crop fields. Studies in China showed that some of these plants, such as the leaves and twigs of *Tephrosia vogelii* do possess strong antifeedant stomach poison and growth inhibiting effects against many insect pests, including the stem borers [47].

Therefore, if the use of ash and other plant derivatives are exploited further and their qualities improved and quantified these will be of great use to the resource-poor farming communities in this region. Most especially if the particular plant is known for its insect control potentials.

## Conclusions

Findings from the survey reveal that farmers in Fako division practiced subsistence agriculture with maize as one of the major crops. The maize is always grown in a mixed cropping system and stem borers are the major field pests limiting attainable yield. The pest burden is greatest during the dry season. Besides maize and other food crops, some farmers also grow cash crops and rare animals for home consumption. Most farmers in the study villages depend on the use of synthetic pesticides to control stem borers. Farmers have limited knowledge of indigenous methods of stem borer control over synthetic, but would prefer it in the future if well informed. This is because, the indigenous and/or plant based pest control methods would be cheaper and readily available as well as being safer if accompanied by standardized methods as well bio-safety and environmental guidelines for efficacy. Farmers would prefer an integrated approach to pest control since it contributes all possible strategies to reduce the pest burden. There was a wide perceptional difference in gender and location similar to most studies. This may differ from scientific studies, but having significant implications for development [32]. To ensure quality and safety, biosafety and quality studies are required for quality assessment of resulting product for human health.

## Abbreviations

ASWA-RUDEP: Association of South West agriculturist rural development project
M: Metre
Asl: Above sea-level
PAN: Pesticide action network
WHO: World Health Organization
PPE: Personal protective equipment
IPM: Integrated pest management
